# Narwhals react to ship noise and airgun pulses embedded in background noise

**DOI:** 10.1098/rsbl.2021.0220

**Published:** 2021-11-10

**Authors:** Outi M. Tervo, Susanna B. Blackwell, Susanne Ditlevsen, Alexander S. Conrad, Adeline L. Samson, Eva Garde, Rikke G. Hansen, Heide-Jørgensen Mads Peter

**Affiliations:** ^1^ Greenland Institute of Natural Resources, Nuuk, Greenland; ^2^ Greenland Institute of Natural Resources, Copenhagen, Denmark; ^3^ Greeneridge Sciences Inc., Santa Barbara, USA; ^4^ Data Science Laboratory, Department of Mathematical Sciences, University of Copenhagen, Copenhagen, Denmark; ^5^ Laboratoire Jean Kuntzmann, University Grenoble-Alpes, Grenoble, France

**Keywords:** narwhal, noise, airgun, arctic, foraging, disturbance

## Abstract

Anthropogenic activities are increasing in the Arctic, posing a threat to niche-conservative species with high seasonal site fidelity, such as the narwhal *Monodon monoceros*. In this controlled sound exposure study, six narwhals were live-captured and instrumented with animal-borne tags providing movement and behavioural data, and exposed to concurrent ship noise and airgun pulses. All narwhals reacted to sound exposure with reduced buzzing rates, where the response was dependent on the magnitude of exposure defined as 1/distance to ship. Buzzing rate was halved at 12 km from the ship, and whales ceased foraging at 7–8 km. Effects of exposure could be detected at distances > 40 km from the ship.At only a few kilometres from the ship, the received high-frequency cetacean weighted sound exposure levels were below background noise indicating extreme sensitivity of narwhals towards sound disturbance and demonstrating their ability to detect signals embedded in background noise. The narwhal's reactions to sustained disturbance may have a plethora of consequences both at individual and population levels. The observed reactions of the whales demonstrate their auditory sensitivity but also emphasize, that anthropogenic activities in pristine narwhal habitats needs to be managed carefully if healthy narwhal populations are to be maintained.

## 1. Introduction

The break-up of sea-ice in the spring as well as calving from glacial fronts and breakdown of icebergs create variable and temporally unpredictable background noise conditions in the Arctic environment that challenge detection and discrimination of acoustic signals [1–3]. Masking of acoustic signals refers to background noise re with the detection of signals of interest, either simultaneously in the frequency domain or in the time domain. Simultaneous masking hinges on the width of the critical band that determines the ability of an individual to discriminate between two nearby frequencies and on the ratio of signal power to noise spectrum level at masked thresholds [4,5]. Directional hearing also plays a role in determining the ability of an animal to localize a sound source in the presence of background noise [6].

Marine mammals use sound for gaining information about their surroundings, including prey, and are, together with echolocating bats, the mammalian groups most specialized to use sound (e.g. [6,7]). Masking studies performed on a handful of species in captivity have demonstrated the extraordinary auditory aptitudes and the complexity of the odontocete sensory system (see [6] for review). Studies of captive whales do not, however, fully address the ability of signal detection in free-ranging whales. Controlled sound exposure studies in the wild in which received sound levels are recorded by animal-borne sensors can be used to determine sound exposure thresholds for behavioural responses. Since the received level at the animal depends on a number of factors including the environment's sound speed profile, and the depth and behaviour of the animal, measuring the received level can be challenging, but studies of behavioural responses can still be used as invaluable indicators of signal detection [8,9].

For the major part of the year, the Arctic is relatively pristine in terms of man-made noise [10,11]. This is changing as a result of a global warming-induced decrease in sea-ice coverage that is making the Arctic more accessible to anthropogenic activities, in both space and time [12–14]. The narwhal, *Monodon monoceros,* is an Arctic toothed whale species that inhabits fjords with erratic ambient noise levels during summer and quieter offshore pack-ice habitats during winter. All studied populations exhibit high-site fidelity towards summer and winter grounds, thereby apparently lacking the plasticity in migratory patterns [15] that is critical for avoiding sustained disturbance. Narwhals must therefore be considered particularly vulnerable to changes in their habitat.

In a controlled sound exposure study, we combined movement and behavioural data from animal-borne tags on narwhals during ship noise and airgun pulse sound exposure trials. We used this information to assess the sensitivity of narwhals to sound exposure in a pristine Arctic soundscape by quantifying sound exposure thresholds for a behavioural response connected to feeding.

## 2. Material and methods

Six male narwhals were live-captured in August 2018 in the Scoresby Sound fjord system in East Greenland in collaboration with local Inuit hunters and instrumented with backpack FastLoc GPS-receivers (Wildlife Computers (Redmond, Seattle, WA, USA) collecting an unrestricted number of FastLoc snapshots through August ([9,15–17] for details on deployment methods and data), and Acousonde™ acoustic and orientation recorders (www.acousonde.com, [18] for details on deployment method) (table 1). Acousondes were set to collect triaxial acceleration and orientation, depth (sampling rate 100 Hz and 10 Hz, respectively), and acoustics. Acoustics were sampled continuously with a 25 811 Hz sampling rate (HTI-96-MIN hydrophone, nominal sensitivity −201 dB re 1 V/μPa, preamp gain 14 dB, an anti-aliasing filter with 3-dB reduction at 9.2 kHz and 22-dB reduction at 11.1 kHz, 16-bit resolution).

**Table 1.** Duration and percentage of observations in distance categories and the number of separate exposures by individual (whale ID). The maximum distance where whales were observed during sound exposure trials was 63 km.

distance category	whale ID	contribution (%)	no. separate exposures
0–20 km (64 h)	B1	13	4
	B2	13	4
	B3	5	2
	B4	13	2
	B5	27	4
	B6	29	6
20–40 km (24.6 h)	B1	15	2
	B2	17	3
	B3	19	1
	B4	7	1
	B5	22	4
	B6	20	3
>40 km (7.4 h)	B1	13	1
	B2	41	1
	B3	19	1
	B4	4	1
	B5	23	1

**Figure 1.**
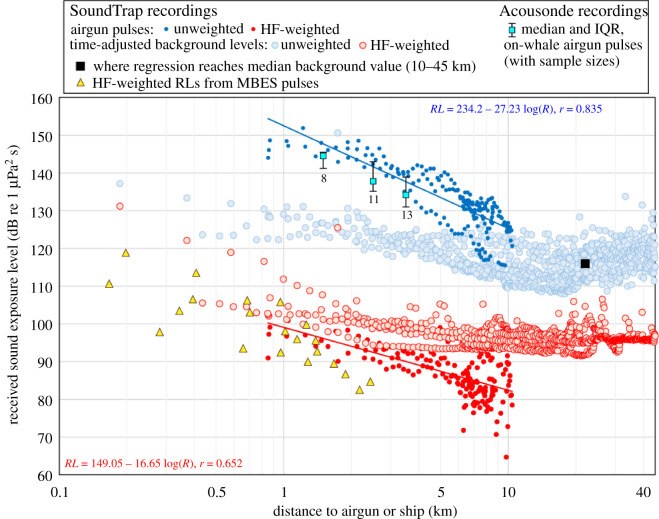
Received SELs of sound from airgun and MBES pulses as compared to background levels, as a function of distance from the sound source. Logarithmic regression fits and their equations are shown for the ST airgun pulse analyses (RL is the received level, *R* is range in *m* and *r* is the correlation coefficient). To enable placing background levels on the same plot as pulse SELs, the 1 s background sample values were adjusted to the mean duration of the airgun pulses (1.34 ± s.d. 0.56 s). This added 10 LOG (1.34) = 1.3 dB to what would have been a 1 s SPL (or SEL) value. See electronic supplementary material for details on analyses [22].

The seismic program was operated from an offshore patrol vessel HDMS *Lauge Koch* equipped with a Reson Seabat 7160 multibeam echo sounder (MBES) (nominal operating frequency 41–47 kHz), that ran continuously. The airgun set-up included a cluster of two Sercel G-guns (17.0 l (1040 in^3^) in total) towed at 6 m depth and operated at a mean pressure of 125 bar. The guns in the cluster were fired synchronously every 80 s during trials, lasting 3–8 h, while the ship's GPS navigation system recorded the location of every shot. Drifting SoundTrap ST202 autonomous recorders (flat frequency response from 20 Hz to 60 kHz, sampling rate 96 kHz, depth 10 m) were used to describe received levels of airgun pulses, ship noise and background noise as a function of range. Background noise levels, measured 10–45 km from the ship, consisted of 1 s samples (10 Hz–48 kHz bandwidth, three-term Blackman–Harris window, NFFT 96000, 50% overlap) selected 3 s before the actual onset of each airgun pulse, as long as the pulses were detectable, and every 80 s thereafter. Airgun pulses were also analysed from Acousonde records on the whales when possible (figure 1; see electronic supplementary material for details on the analyses; see [9] for more information).

Time–depth records were down-sampled to 1 Hz and time-synchronized with GPS positions. Additional GPS positions were created for each second between successive positions through linear interpolation [9]. Buzzes were used as a proxy for foraging attempts [6] and were detected from the Acousonde acoustic data using a custom-written detector (Matlab, The MathWorks Inc., USA) and verified manually.

When the sound source and animal were within line of sight (determined visually from maps showing the positions of the ships and whales aligned in time), distance between the whale and the sound source was determined for each second. Exposure was defined as 1/distance to ship (in km) resulting in higher exposure with decreasing distance to the ship. Exposure was denoted zero before the experiment began representing undisturbed behaviour. The effect of exposure on the buzzing rate (presence/absence of buzz start at 1 s time bins) was modelled using a generalized linear mixed model in R [19] (glmer, package *lme4,* [20]) with a Poisson response distribution with a log-link, where exposure was entered nonlinearly as an explanatory variable using natural cubic splines with three degrees of freedom (ns, package *splines*) with internal knots located at the 33th and 66th percentiles of the non-zero exposure values. Individual was included as a random effect allowing each animal to have a unique baseline (intercept) in their sound production rate. Moreover, the model included an autoregressive memory component of order 63 s to account for autocorrelation in the buzzing activity [21]. Details of the model and model testing are specified in the electronic supplementary material [22].

## 3. Results and discussion

A log fit on received sound exposure levels (SELs) of airgun pulses reached the median background noise level, 115.9 dB re 1 µPa^2^ s, 22.1 km from the sound source (figure 1). Received levels of airgun pulses (*n* = 32) measured from whale-borne Acousonde recorders (*n* = 3), 1–4 km from the airgun, showed reasonable overlap with levels obtained from ST recordings (figure 1). High-frequency cetacean (HF) weighting [23], which provides a better estimate of actual levels perceived by the whales, lowered the airgun pulse SELs and background noise SELs less than 10 km by 28–61 and 9–32 dB, respectively, compared with unweighted values (figure 1). Near the ship, HF-weighted background SELs approached unweighted values (minimum difference was 6 dB) in part due to the presence of the MBES signals (figure 1, see [9] for more details). Within approximately 3 km of the source, MBES signals were therefore part of the sound exposure the whales were experiencing, but beyond this distance, the whales were presumably reacting to a combination of airgun pulses and ship noise (figure 1).

**Figure 2.**
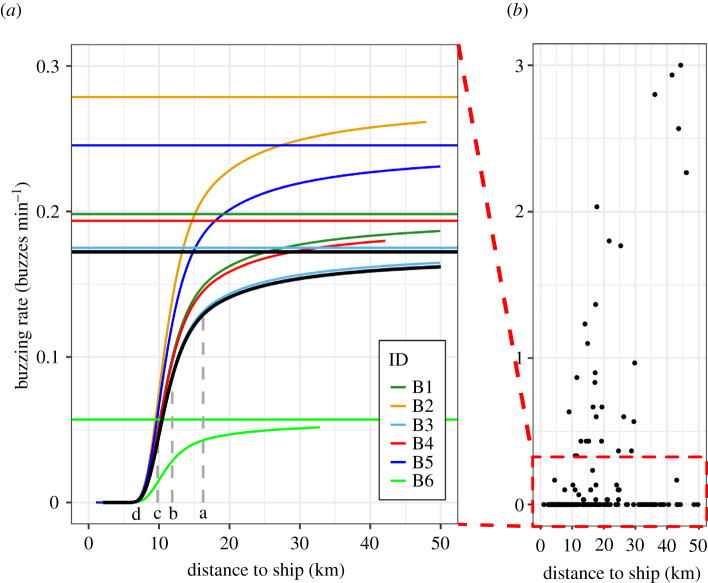
The effect of sound exposure on the buzzing rate as a function of distance to the sound source as model outputs (*a*) and raw data (*b*). The curved black line indicates the population-level estimate of the effect and the horizontal black line indicates the undisturbed buzzing rate on a population level. Individual estimates and the corresponding undisturbed buzzing rates are given in different colours. The vertical grey dashed lines indicate the distances (*a*–*d*) at which the population-level buzzing rate decreased by 25%, 50%, 75% and 100% as an effect of exposure (table 2).

The six male narwhals in this study showed clear behavioural responses to the exposure of concurrent ship noise, MBES pulses and airgun pulses, with a significant effect on the buzzing rate (*p* < 0.0001; table 2 and figure 2).

**Table 2.** Distances from the sound source (km) at which, compared with undisturbed behaviour, there was a population-level decrease of 25%, 50%, 75% and 100% in the buzzing rate during sound exposure trials i.e. ship noise and airgun pulses (*a*–*d*, figure 2). The estimated SEL at these distances are given both as unweighted and HF-weighted [23] values (figure 1). The cells highlighted with grey represent ranges where the computed SELs that the whales were reacting to were below background noise level. The values in the grey cells indicate the maximum background levels measured at these ranges. The interquartile range of background levels at these ranges were 113–119 dB re 1 µPa^2^-s in unweighted data and 95–97 dB re 1 µPa^2^ s in HF-weighted data. Background levels were adjusted to the mean duration of the airgun pulses (1.34 ± s.d. 0.56 s, figure 1).

decrease in buzzing rate (%)	distance to sound source (km)	unweighted SEL (dB re 1 μPa^2^ s)	HF-weighted SEL (dB re 1 μPa^2^ s)
25	16 (a)	<134	<107
50	12 (b)		
75	10 (c)		
100	approximately 7–8 (d)	<135

All individuals ceased foraging within approximately 7–8 km of the ship at received HF-weighted airgun pulse SELs below background noise levels of 107 dB (interquartile range 95–97 dB) re 1 μPa^2^ s (figure 2 and table 2). At this distance, noise from the ship was also buried in the background, but was difficult to quantify (see [9]); the unweighted airgun pulse SELs were less than 135 dB re 1 μPa^2^ s (table 2). Compared with undisturbed behaviour, a 25% and 50% decrease in buzzing rate occurred at 16 and 12 km from the source, respectively (figure 2). At these distances, the estimated received SELs—both unweighted and HF-weighted—were below background levels, further demonstrating the ability of the whales to detect signals embedded in background noise (table 2). Each of the six individuals, during independent trials, contributed 5–29% of the 64 h of data in range-category ‘0–20 km’ (table 1) supporting that the modelled reduction in buzzing rate predicts a true behavioural response within the population (figure 2).

The effect of sound exposure on buzzing rate could be detected out to the range-category ‘greater than 40 km’ (figure 2). This category, however, only represented 7.4 h of data and individual B2 contributed almost half of that duration (table 1). The response at these remote distances may therefore be driven by individual variation, spatial or behavioural context, and can be used as a proof of sensitivity only in a limited context. Although our data cannot be used to determine signal detection range in narwhals in the Scoresby Sound fjord system, our results imply detection at ranges greater than 40 km from the source. Narwhals have been shown to react to icebreaker noise at greater than 55 km in Lancaster Sound [24,25]. Although the acoustic environment in northern Baffin Bay is different from Scoresby Sound, the observations corroborate our finding of narwhals reacting to low SELs.

Other studies have found that exposure to airgun pulses at levels of 146 and 162 dB re 1 µPa (*p*–p) and 131 dB re 1 µPa^2^ s (SEL) did not elicit observable reactions in sperm whales *Physeter macrocephalus,* neither in a semi-pristine high latitude habitat nor in a highly trafficked area, respectively [26,27], possibly implying robustness towards disturbance by this species. In the other extreme are the beaked whales *Ziphiidae* sp., which are regarded as one of the most sensitive cetaceans to sound disturbance. They have been shown to decrease or cease foraging as a reaction to low sonar signal levels ranging between 98 and 140 dB re 1 µPa (unweighted) [28–30]. Also harbour porpoises *Phocoena phocoena* have been shown to react to high-frequency ship noise at SPL levels as low as 98 dB re 1 µPa by reduced feeding [31]. Although direct comparisons between SPLs eliciting responses in these studies are not valid due to different signal types, our results of reduced foraging are comparable, placing narwhals among the most sensitive cetaceans to sound disturbance.

## 4. Conclusion

This study showed narwhals to be highly sensitive to anthropogenic noise. The whales clearly reacted to sound disturbance embedded in the highly variable background noise of their environment, as far as greater than 40 km from the sound source, by first reducing, then eliminating their buzzing activity. This likely leads to reduced foraging success, and will, if combined with sustained disturbance over longer periods, have energetic costs at the population level. If healthy, undisturbed narwhal populations are to be maintained, the whales' extreme sensitivity to man-made sounds needs to be considered when assessing and regulating anthropogenic activities in the Arctic.

## Data accessibility

Data on exposure and behavioural responses of narwhals: Dryad https://doi.org/10.5061/dryad.000000046 [17]. The data are provided in the electronic supplementary material [22].
